# The DNA methylation profile of liver tumors in C3H mice and identification of differentially methylated regions involved in the regulation of tumorigenic genes

**DOI:** 10.1186/s12885-018-4221-0

**Published:** 2018-03-22

**Authors:** Junya Matsushita, Kazuyuki Okamura, Kazuhiko Nakabayashi, Takehiro Suzuki, Yu Horibe, Tomoko Kawai, Toshihiro Sakurai, Satoshi Yamashita, Yoshikazu Higami, Gaku Ichihara, Kenichiro Hata, Keiko Nohara

**Affiliations:** 1 Center for Health and Environmental Risk Research, National Institute for Environmental Studies, Tsukuba, Japan; 2 Graduate School of Pharmaceutical Sciences, Tokyo University of Science, Noda, Japan; 3 Department of Maternal-Fetal Biology, National Center for Child Health and Development, Tokyo, Japan; 4 National Cancer Center Japan, Tokyo, Japan

**Keywords:** C3H mice, DNA methylation, Liver tumors, Reduced representation bisulfite sequencing (RRBS), 5-aza-2'-deoxycytidine

## Abstract

**Background:**

C3H mice have been frequently used in cancer studies as animal models of spontaneous liver tumors and chemically induced hepatocellular carcinoma (HCC). Epigenetic modifications, including DNA methylation, are among pivotal control mechanisms of gene expression leading to carcinogenesis. Although information on somatic mutations in liver tumors of C3H mice is available, epigenetic aspects are yet to be clarified.

**Methods:**

We performed next generation sequencing-based analysis of DNA methylation and microarray analysis of gene expression to explore genes regulated by DNA methylation in spontaneous liver tumors of C3H mice. Overlaying these data, we selected cancer-related genes whose expressions are inversely correlated with DNA methylation levels in the associated differentially methylated regions (DMRs) located around transcription start sites (TSSs) (promoter DMRs). We further assessed mutuality of the selected genes for expression and DNA methylation in human HCC using the Cancer Genome Atlas (TCGA) database.

**Results:**

We obtained data on genome-wide DNA methylation profiles in the normal and tumor livers of C3H mice. We identified promoter DMRs of genes which are reported to be related to cancer and whose expressions are inversely correlated with the DNA methylation, including *Mst1r*, *Slpi* and *Extl1*. The association between DNA methylation and gene expression was confirmed using a DNA methylation inhibitor 5-aza-2′-deoxycytidine (5-aza-dC) in Hepa1c1c7 cells and Hepa1-6 cells. Overexpression of *Mst1r* in Hepa1c1c7 cells illuminated a novel downstream pathway via IL-33 upregulation. Database search indicated that gene expressions of *Mst1r* and *Slpi* are upregulated and the TSS upstream regions are hypomethylated also in human HCC. These results suggest that DMRs, including those of *Mst1r* and *Slpi,* are involved in liver tumorigenesis in C3H mice, and also possibly in human HCC.

**Conclusions:**

Our study clarified genome wide DNA methylation landscape of C3H mice. The data provide useful information for further epigenetic studies of mice models of HCC. The present study particularly proposed novel DNA methylation-regulated pathways for *Mst1r* and *Slpi*, which may be applied not only to mouse HCC but also to human HCC.

**Electronic supplementary material:**

The online version of this article (10.1186/s12885-018-4221-0) contains supplementary material, which is available to authorized users.

## Background

C3H mice have been employed for numerous studies of carcinogenesis. The mice, particularly their males, are predisposed to spontaneously develop liver tumors in adulthood and have been used as an animal model of spontaneous hepatocellular carcinoma (HCC) [1, 2]. They are also often used as chemically induced HCC models, such as diethylnitrosamine (DEN)- or DEN and phenobarbital (DEN/PB)-induced HCC models [1, 3–5]. A recent study established an HCC model with liver cirrhosis, a key feature of human HCC, by administering carbon tetrachloride (CCl_4_) to C3H mice [6].

To investigate the process of carcinogenesis and therapeutic methods in proper animal models, information on the genetic and epigenetic background of their tumors should greatly help understanding the mechanisms involved. A well-known genetic feature of spontaneous liver tumors of C3H mice is the occurrence of somatic mutations in the proto-oncogene Ha-*ras* in approximately 10–60% of the tumors [2]. Somatic point mutations of *ras* genes (Ha-*ras*, K-*ras*, and N-*ras*) were also detected in a variety of human and animal tumors^7^. These somatic mutations activate Ras proteins and lead to activation of tumor augmenting pathways, including the RAF/MEK/ERK kinase cascade [7]. Another somatic mutation was reported in B-*raf*, a member of the RAF kinase family in about 20% of liver tumors in C3H mice administered DEN at 2 weeks of age [8]**.** B-*raf* mutation also augments the kinase activity leading to activation of the MEK/ERK cascade [5, 8].

On the other hand, some of the spontaneous liver tumors of C3H mice do not have Ha-*ras* or B-*raf* mutations [8, 9]. Epigenetic modifications, such as DNA methylation and histone modifications, are pivotal posteriori control mechanisms of gene expression and are closely involved in tumorigenesis [10]. A recent study performed next-generation sequencing of DNA methylation of human non-cirrhotic HCC and fibrolamellar hepatocellular carcinoma (FLC) and found distinctive epigenetic signatures of the two types of tumors [11]. While we previously studied methylation status in the spontaneous liver tumors of control C3H mice and liver tumors of gestationally arsenite exposed C3H mice by a methylated DNA immunoprecipitation (MeDIP)–CpG island microarray method [12], precise single base resolution analysis of DNA methylation has not been performed for the liver tumors of C3H mice.

Recent progress in genomic study using next generation sequencing enabled DNA methylation analysis at one-base pair resolution and revealed more precise modes of action of DNA methylation, beyond gene silencing by canonical CpG islands. DNA methylation is involved in suppression/activation of gene expression, transcription factor binding, splicing and nucleosome posioning [13]. In the present study, we clarified differentially methylated CpGs (DMCs) and differentially methylated regions (DMRs), where DNA in the spontaneous liver tumors of C3H mice is hyper- or hypomethylated compared to the normal livers, by reduced representation bisulfite sequencing (RRBS) method, a sequencing-based genome-wide DNA methylation analysis [14, 15]. According to the recent reports showing that DNA methylation levels within ± 1000 bp regions [13] or ± 2000 bp regions [11, 16, 17] of TSS showed a strong correlation with gene repression, we focused on DMRs within ± 2000 bp of TSS (promoter DMRs). Using the data of promoter DMRs and by in vitro experiments and PubMed search, we identified genes whose expressions are closely associated with the methylation of proximal promoter DMRs and which are involved in tumorigenesis. We also assessed the gene expression and DNA methylation status of these genes in human HCC using TCGA database to explore similarities in tumorigenic regulation in human.

## Methods

### Animals

Male C3H/HeN mice around 74 weeks of age were obtained from F2 pups of the control group of a project for investigating the effects of gestational arsenic exposure on the F2. The design of animal breeding was the same as described previously [9, 18]. Briefly, pregnant F0 C3H/HeN mice were purchased from CLEA Japan (Tokyo, Japan) and F1 and F2 pups in the control group were given free access to a standard diet (CA-1; CLEA Japan) and tap water (the control group). The mice were handled in a humane manner in accordance with the National Institute for Environmental Studies (NIES) guidelines for animal experiments.

### Cell lines and treatment

Hepa1c1c7 cells were kindly provided by Dr. Y. Fujii-Kuriyama (University of Tsukuba) in 2005 [19] and cultured in the Dulbecco’s Modified Eagle’s Medium (DMEM, SIGMA-ALDRICH, D5796) containing 10% FBS and 1% penicillin-streptomycin. Hepa1-6 cells provided by RIKEN BRC CELL BANK were cultured in DMEM containing 10% FBS, 1% penicillin-streptomycin and 5% sodium pyruvate. 5-Aza-dC was purchased from Santa Cruz (CA, USA). After attaching to the dish, cells were incubated in a culture medium containing 5-aza-dC for 72 h. For overexpression study, mouse ORF clones Mst1r (NM_001287261) and Slpi (NM_011414), and pCMV6-Kan/Neo (pCMV6KN) as an empty vector were purchased from ORIGENE Inc. (MD, USA). Hepa1c1c7 cells were transfected with 2 μg of vectors using HilyMax (Dojindo, Japan) according to the manufacturer’s instructions and gene expression changes were examined after culturing for 24 h.

### Genomic DNA extraction

Genomic DNA was prepared as previously described [18] from the normal livers and macroscopic liver tumor tissues of C3H mice and cell lines. Briefly tissues or cells were lysed in lysis buffer, treated with RNase, and purified with a phenol-chloroform mixture.

### RRBS analysis

RRBS libraries were prepared from genomic DNA of 3 normal liver tissues and 3 tumor tissues having a Ha-*ras* mutation according to the protocol reported by Boyle et al. [15]. with some modifications. Briefly, 100 ng of genomic DNA was digested with MspI, subjected to gap-filling and A-tailing, and ligated with TruSeq adaptors included in the TruSeq DNA Sample Prep Kit (FC-121-1001, Illumina). After DNA size selection and ligation efficiency were checked, adaptor-ligated DNA was subjected to bisulfite conversion and amplified by PCR. The PCR products were cleaned-up using Agencourt AMPure XP beads and sequenced on an Illumina HiSeq2500. The sequence data have been deposited in NCBI’s Gene Expression Omnibus and are accessible through GEO Series accession number GSE111420 (http://www.ncbi.nlm.nih.gov/geo/query/acc.cgi?acc=GSE111420).

### Alignment of RRBS data and identification of DMCs and DMRs

Bisulfite-treated sequencing reads were aligned by a paired-end alignment method for a unique best hit to the mouse reference genome (NCBI/mm10) using the Bismark program [20], and adapter trimming and quality control was performed using Trim Galore (http://www.bioinformatics.babraham.ac.uk/projects/trim_galore/). The aligned data were used for generating and analyzing DNA methylation profiles using the methylKit package [21]**.** Briefly, %methylation scores were determined for CpG sites using the *read.bismark* function of methylKit as SAM format alignments files created with the Bismark aligner as input files. For each CpG site, the *read.bismark* function counts the numbers of reads with C (methylated and remained) and with T (unmethylated and converted) in both strands separately, and calculates %methylation. The obtained %methylation profiles were evaluated for their overall statistics and extent of overall similarities among samples using methyKit functions such as *getMethylationStats* and *getCorrelation* [21].

DMCs were extracted using methylKit for the cytosines in CpG sites with < 0.01 q-value by the logistic regression method, ≥10 reads in coverage and ≥ 25% methylation differences between normal and tumor tissues. Under the condition, 2,331,643 CpG sites were identified. DMRs were detected for regions containing ≥3 CpG sites and at least 1 DMC and with ≥30% absolute mean CpG methylation difference by using an eDMR package on R. eDMR implements statistical analysis [22]. Promoter DMRs, which were defined as regions within ±2000 bp from TSSs for Refseq transcripts [11, 16, 17], were extracted using bedtools 2.25.0 (bedtools.readthedocs.io). A summary of the detailed sequencing and alignment characteristics for the samples used in this study will be published elsewhere with the data of arsenite-exposed F2 mice (Okamura et al., in preparation).

### Bisulfite sequencing

Bisulfite sequencing analysis was carried out as previously described with minor modifications [23]. Genomic DNA (1 μg) was digested with EcoRI and subjected to bisulfite modification using EZ DNA Methylation-Gold Kit (Zymo Research, CA, USA). The bisulfite-treated DNA (2 ng) was amplified with the primers shown in Additional file 1: Table S1. The PCR products were purified with the Wizard SV Gel and PCR Clean-Up System (Promega) and cloned into pGEM-T Easy Vector (Promega). Obtained clones were cycle sequenced with M13RV primers (5′- CAGGAAACAGCTATGAC-3′) and a BigDye Terminator version 3.1 Cycle Sequencing Kit (Applied Biosystems, CA, USA), and analyzed with an Applied Biosystems 3730 DNA analyzer.

### Microarray analysis of gene expression

Total RNA was prepared from the 3 normal liver tissues, 3 tumor tissues having Ha-*ras* mutation and Hepa1c1c7 cells with an RNeasy Mini Kit (Qiagen, Valencia, CA, USA). After the quality of total RNA was checked using a 2100 Bioanalyzer (Agilent Technologies), the gene expression profile was analyzed using SurePrint G3 Mouse GE arrays (Agilent Technologies) according to the manufacturer’s protocol for one-color microarray-based gene expression analysis. The scanned images were analyzed with Feature Extraction Software 9.1 to obtain background subtracted and spatially detrended Processed Signal intensities. The microarray data have been deposited in NCBI’s Gene Expression Omnibus and are accessible through GEO Series accession number GSE104627 (https://www.ncbi.nlm.nih.gov/geo/query/acc.cgi?acc=GSE104627) and GSE104626 (https://www.ncbi.nlm.nih.gov/geo/query/acc.cgi?acc=GSE104626).

The expression data from each chip were then normalized to the 75the percentile of all measurement using Agilent Gene-Spring GX. Genes with an expression value of more than 1.0, in either normal tissues or tumor tissues or in both, were considered to be expressed above the threshold and utilized for analysis. The genes with a two- or more fold increase or decrease in mean expression values (*n* = 3) were judged to have changed. For Hepa1c1c7 cells with or without overexpression of *Mst1r* or *Slpi*, genes with raw expression values of more than 50, either after upregulation or before downregulation or in both cases, were utilized for further analysis.

### Real-time PCR analysis

Total RNA was prepared from tissues and cells with an RNeasy Mini Kit (Qiagen). cDNA synthesis and real-time PCR was carried out as described previously [24]. The primers used are shown in Additional file 1: Table S1.

### Prediction of the transcription factor binding site

Consensus regions of transcription factor binding sites were detected using the JASPAR database (http://jaspar.genereg.net/) [25] with the relative score threshold set at > 90% and also by using published literature.

### TCGA analysis

Forty one paired datasets of normal and HCC tissues of human livers for RNA-seq and DNA methylation levels were obtained from the TCGA database (http://cancergenome.nih.gov/). They were downloaded and used to find genes with significant expression differences and differences in DNA methylation within ±2000 bp from TSS between normal and HCC tissues.

### Statistical analysis

The differences in gene expression between the two groups were analyzed by a two-tailed paired Student’s t-test. A *p*-value < 0.05 was considered to be statistically significant. The differences in gene expression among three groups were analyzed by one-way ANOVA followed by the Turkey-Kramer test as a post hoc comparison. Spearman correlation analysis was used to determine the relationship between DNA methylation differences and gene expression ratios of normal and liver tumors in C3H mice. For multiple comparisons to identify DNA methylation differences, paired t-test with Bonferroni correction was performed.

## Results

### Genome wide analysis of DNA methylation and gene expression in normal and tumor livers of C3H mice

**Fig. 1:**
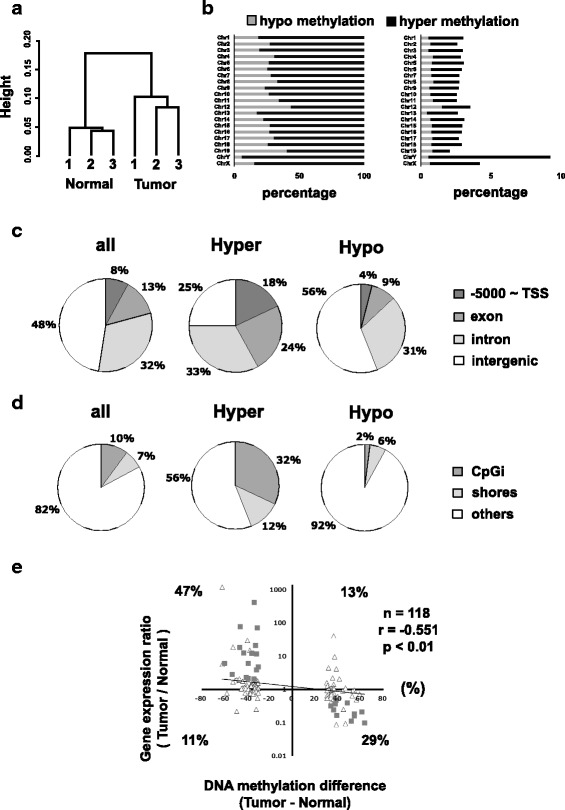
Outline of DNA methylation status of normal and tumor liver tissues in C3H mice. **a** Hierarchical clustering of methylation profiles of normal and tumor tissues using 1-Peason’s correlation distance. **b** Horizontal bar plots showing the percentage of hyper- and hypo-DMCs in all DMCc (left) and in all CpG (right) per chromosome. **c** Pie charts showing percentage of DMCs in the region between − 5000 bp and TSS, exon, intron and intergenic. **d** Pie charts showing percentage of DMCs in CpG islands, CpG island shores (defined as 2 kb flanks of CpG islands) and other regions. **e** The scatter plot of DNA methylation differences of DMRs and gene expression ratios between the normal and tumor tissues. The data of 118 expressing genes with the promoter DMRs were used. The relationship between DNA methylation differences and gene expression ratios of normal and liver tumors was determined by Spearman correlation analysis. 28 DMRs whose associated genes are up- or downregulated by hypo- or hyper-DMRs more than 2-fold in tumor tissues and appear in a PubMed search for the term “cancer” were shown in square, and listed in Table 1. Triangle plots indicate other DMRs

We conducted genome wide DNA methylation analysis for normal liver tissues and tumor tissues of male C3H mice by RRBS. Approximately 22–55 million sequence read pairs were obtained for the RRBS libraries and 63–71% of the reads were aligned to unique genomic locations. Hierarchical clustering analysis showed that DNA methylation status was distinctly separated between the normal and tumor tissues (Fig. 1a). Scatter plots of methylation values show high correlation coefficient value for each pair (Additional file 2: Figure S1).

We identified 103,965 DMCs (4.5% in total CpGs) with higher ratios of hypo-methylated CpGs in tumor tissues (hypo-DMCs), such as 60–80% in total DMCs in autosomal chromosomes and more than 90% in sex chromosomes (Fig. 1b). Annotation analysis showed that the majority of DMCs were in intron and intergenic regions (Fig. 1c) and in the region other than CpG island (CGI) or CGI shore (Fig. 1d). Higher ratio of hyper-DMCs, compared with hypo-DMCs, were found in the region between − 5000 bp upstream and TSS (Fig. 1c) and in CGI and CGI shore (Fig. 1d).

We then identified 3337 DMRs (Additional file 3: Table S2) including 225 promoter DMRs. Among the 225 genes driven by the differentially methylated promoters, 118 genes are expressed (see Methods). The methylation status of the 118 promoter DMRs was inversely associated with gene expression [13, 16, 17]. The scatter plot of DNA methylation differences of DMRs and gene expression ratios between the normal and tumor tissues of the 118 genes showed moderate inverse correlation (Fig. 1e). From the 118 genes, we shortlisted 27 genes which show more than 2-fold expression differences (*p* < 0.05) between normal and tumor tissues and inverse association with methylation status of the DMR, and which appear in a PubMed search for the term “cancer” (Table 1 and gray square in Fig. 1e).

**Table 1:** Shortlist of the selected genes^a^

Associated gene	discription	DMR position	DMR Distance from TSS	DMCs number	Methylation difference (Tumor -Normal)	Gene expression (Tumor / Normal)	
						ratio	*p*-value
downstream							
Slpi	secretory leukocyte protease inhibitor	chr2: 164355520–164,355,812	+ 697	9	−33.83	408.37	0.0090
Cela1	chymotrypsin-like elastase family, member 1	chr15: 100687184–100,687,240	+ 682	3	−31.23	71.51	0.0003
Mst1r	macrophage stimulating 1 receptor	chr9: 107908081–107,908,516	+ 1192	13	−46.34	67.47	0.0005
Btg2	B cell translocation gene 2	chr1: 134077506–134,077,566	+ 1591	3	−46.61	18.30	0.0050
Fgl2	fibrinogen-like protein 2	chr5: 21373036–21,373,583	+ 366	10	−31.73	11.88	0.0002
P2rx7	purinergic receptor P2X	chr5: 122644473–122,644,655	+ 562	4	−43.12	8.23	0.0031
Lss	lanosterol synthase	chr10: 76532531–76,532,879	+ 904	8	−59.93	6.17	0.0000
Grhl1	grainyhead-like 1	chr12: 24573292–24,573,626	+ 1005	3	−39.22	6.06	0.0020
Itm2c	integral membrane protein 2c	chr1: 85895741–85,895,786	+ 1231	3	−31.78	3.91	0.0015
Chmp6	charged multivesicular body protein 6	chr11: 119915542–119,915,667	+ 1732	6	−53.11	2.88	0.0002
Vasp	vasodilator-stimulated phosphoprotein	chr7: 19269155–19,269,927	+ 1929	14	−40.96	2.27	0.0016
Mmp14	matrix metallopeptidase 14	chr14: 54433543–54,434,231	**+** 1939	13	−39.11	2.19	0.0012
Marveld2	MARVEL domain containing 2	chr13: 100615630–100,615,803	+ 1143	2	−34.10	2.06	0.0238
Pdk2	pyruvate dehydrogenase kinase, isoenzyme 2	chr11: 95039447–95,039,657	+ 1716	7	38.16	0.48	0.0034
Extl1	exostoses-like 1	chr4: 134371099–134,371,620	+ 929	44	48.12	0.39	0.0216
Grm8	glutamate receptor, metabotropic 8	chr6: 28133156–28,133,165	+ 1206	3	33.66	0.32	0.0016
Ppp1r14a	protein phosphatase 1, regulatory inhibitor subunit 14A	chr7: 29291240–29,291,527	+ 1920	8	36.03	0.25	0.0052
Snx29	sorting nexin 29	chr16: 11405750–11,405,827	+ 102	3	60.92	0.22	0.0218
C8b	complement component 8, beta polypeptide	chr4: 104767260–104,767,325	+ 943	7	54.51	0.18	0.0066
Igfals	insulin-like growth factor binding protein, acid labile subunit	chr17: 24880338–24,882,131	+ 1568	57	38.73	0.17	0.0003
Adam11	a disintegrin and metallopeptidase domain 11	chr11: 102762268–102,762,503	+ 829	16	54.16	0.16	0.0002
Cyp8b1	cytochrome P450, family 8, subfamily b, polypeptide 1	chr9: 121914943–121,915,025	+ 1279	6	63.26	0.10	0.0231
Upstream							
Ltb	lymphotoxin B	chr17: 35194367–35,194,502	−4	3	−32.55	20.94	0.0410
Nfe2	nuclear factor, erythroid derived 2	chr15: 103259509–103,259,588	− 1105	4	−36.04	12.39	0.0045
Hmgcr	3-hydroxy-3-methylglutaryl-coenzyme A reductase	chr13: 96671738–96,671,790	− 801	1	−30.40	4.80	0.0144
Slc29a1	solute carrier family 29 member 1	chr17: 45600404–45,600,454	− 800	2	37.50	0.37	0.0010
Slc17a8	solute carrier family 17	chr10: 89621306–89,622,039	−60	7	51.72	0.11	0.0142

^a^These genes are hit by PubMed search for the term “cancer” and whose expressions are up- or down-regulated more than 2-fold in an inverse manner compared to the methylation rates between the normal and tumor tissues. Chr: chromosome, DMR: differentially methylated region, DMC: differentially methylated cytosine, TSS: transcription start site. *P*-values were analyzed by Student’s t-test

### Expressions of *Mst1r, Slpi* and *Extl1* are associated with DNA methylation alterations in the liver tumors of C3H mice

Among the genes shown in Table 1, we focused on *Mst1r* and *Slpi,* which are greatly up- regulated (67- and 408-fold, *p*-value 0.0005 and 0.009, respectively) and have relatively large numbers of DMCs (13 and 9) in the DMRs, and *Extl1*, which have a large number of DMCs (44 DMCs) in the DMR. Mst1r is a receptor tyrosine kinase activated by the binding of the ligand, Mst1 (macrophage-stimulating 1, also known as hepatocyte growth factor-like; HGFL) [26, 27]. Overexpression of Mst1r activates the molecule without the need of the ligands [27]. Such overexpression and activation of Mst1r are found in many types of human cancers and often correlate with poor prognosis in various cancers [27]. Slpi has activity as serine protease inhibitor and is overexpressed in various types of cancers [28], including mouse liver tumors induced by CCl_4_ or DEN [4]. *Extl1* is a putative tumor-suppressor gene found in neuroblastoma patients [29].

**Fig. 2:**
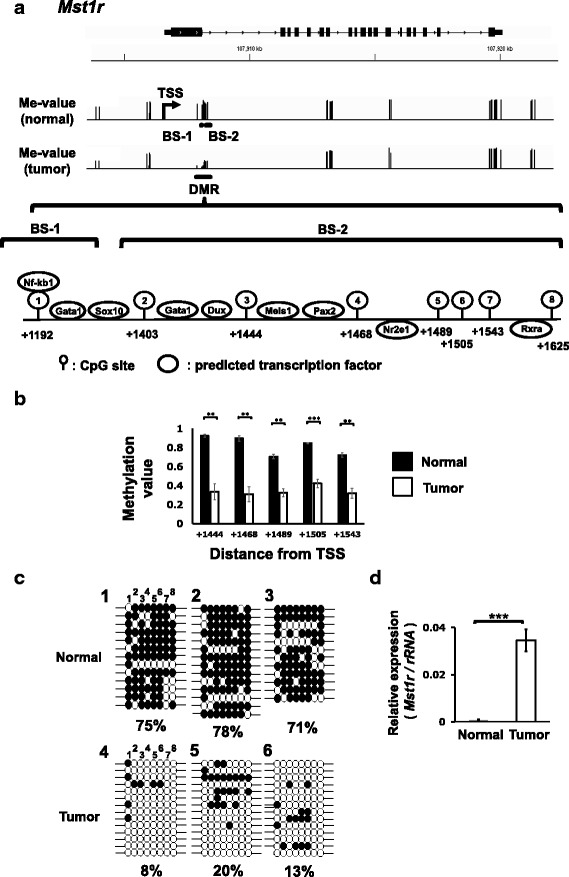
*Mst1r* associated with hypo-DMR is up-regulated in the liver tumor tissues of C3H mice. **a** Schematic view of the *Mst1r* locus. Methylation levels of CpG sites were visualized using Integrative Genomics Viewer (IGV) software. The position of TSS, DMR and the positions assessed by bisulfite sequencing (BS-1 and BS-2) were indicated. The magnified view indicates 8 CpG positions in the DMR and predicted transcription factor binding sites. **b** The average methylation values of the CpG sites in normal and tumor tissues. The CpG sites with ≥10 reads on both strands (≥20 reads in total) are indicated. Statistical significance between normal and tumor tissue was analyzed by Student’s t-test. **, *** Significantly different at *p* < 0.01 and 0.001, respectively. **c** Validation of RRBS data for normal and tumor liver tissues of C3H mice by bisulfite sequencing. Methylated and unmethylated cytosine are shown as ● and ○ respectively. The numbers above the circles indicate the position of CpG shown in Fig. 1a. **d** Validation of gene expression levels of *Mst1r* by real-time PCR in the normal (*n* = 6) and tumor tissues (*n* = 11) of C3H mice. The expression of *Mst1r* is normalized to the expression of *rRNA*. Statistical significance between the two groups was analyzed by Student’s t-test. *** Significantly different at *p* < 0.001

**Fig. 3:**
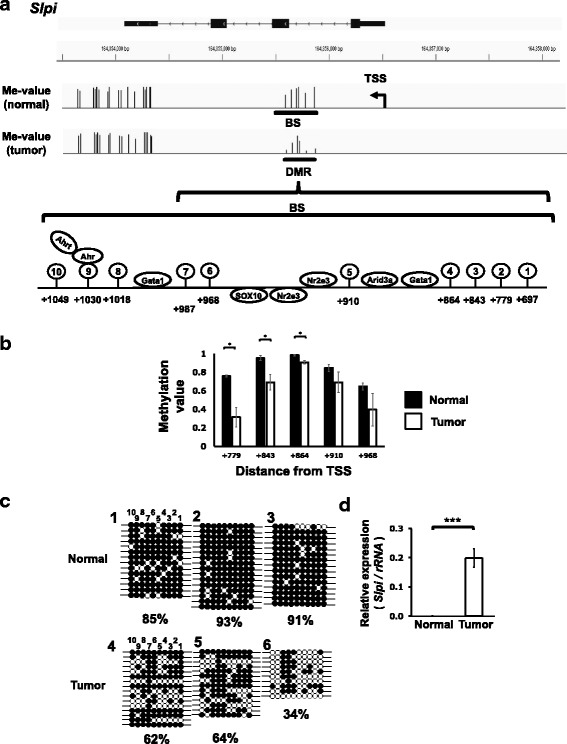
*Slpi* associated with hypo-DMR is up-regulated in the liver tumor tissues of C3H mice. **a** Schematic view of the *Slpi* locus. The position of TSS, DMR and the position assessed by bisulfite sequencing (BS) were indicated. The magnified view indicates 7 CpG positions in the DMR and predicted transcription factor binding sites. **b** The average methylation values of the CpG sites in normal and tumor tissues. The CpG sites with ≥10 reads on both strands are indicated. Statistical significance between normal and tumor tissue was analyzed by Student’s t-test. * Significantly different at *p* < 0.05. **c**Validation of RRBS data for normal and tumor liver tissues of C3H mice by bisulfite sequencing. Methylated and unmethylated cytosine are shown as ● and ○ respectively. **d** Validation of gene expression levels of Slpi by real-time PCR in the normal (*n* = 6) and tumor tissues (*n* = 11) of C3H mice. The expression of Slpi is normalized to the expression of *rRNA*. Statistical significance between the two groups was analyzed by the Student’s t-test. *** Significantly different at *p* < 0.001

**Fig. 4:**
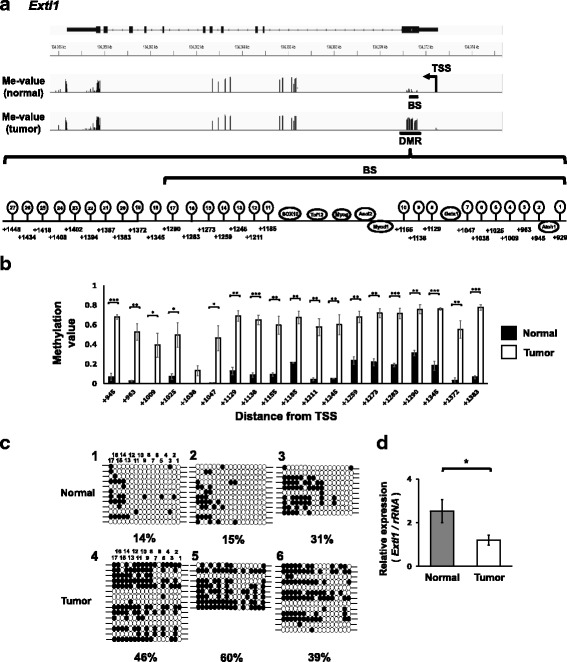
*Extl1* associated with hyper-DMR is down-regulated in the liver tumor tissues of C3H mice. **a** Schematic representation of the *Extl1* locus. The position of TSS, DMR and the position assessed by bisulfite sequencing (BS) were indicated. The magnified view indicates 27 CpG positions in the DMR and predicted transcription factor binding sites. **b** The average methylation values of the CpG sites in normal and tumor tissues. The CpG sites with ≥10 reads on both strands are indicated. Statistical significance between normal and tumor tissue was analyzed by Student’s t-test. *, **, *** Significantly different at *p* < 0.05, 0.01 and 0.001, respectively. **c** Validation of RRBS data for normal and tumor liver tissues of C3H mice by bisulfite sequencing. Methylated and unmethylated cytosine are shown as ● and ○ respectively. **d** Validation of gene expression levels of *Extl1* by real-time PCR in the normal (*n* = 6) and tumor tissues (*n* = 11) of C3H mice. The expression of *Extl1* is normalized to the expression of *rRNA*. Statistical significance between the two groups was analyzed by the Student’s t-test. * Significantly different at *P* < 0.05

The DNA methylation status of these genes are visualized in Figs. 2a, 3a, and 4a. *Mst1r* and *Slpi*, which are upregulated in the tumors, have hypo-DMRs starting from + 1192 and + 697 bps from TSS, respectively. *Extl1,* whose expression is downregulated in the tumors, has hyper-DMR starting from + 929 bp from TSS. We validated the DNA methylation status of these DMRs by bisulfite sequencing (Figs. 2b, 3b, and 4b) and confirmed that the RRBS data correlated well with the bisulfite sequencing data. We also measured gene expression levels of the normal and tumor tissues by real-time PCR and confirmed up-regulation of *Mst1r* and *Slpi* and down-regulation of *Extl1* in the tumor tissues (Figs. 2c, 3c, and 4c). The expression levels of these genes did not differ between tumor tissues with and without Ha-*ras* mutation (Additional file 2: Figure S2).

### Expressions of *Mst1r, Slpi*, and *Extl1* are upregulated in cancer cell lines by 5-aza-dC treatment

**Fig. 5:**
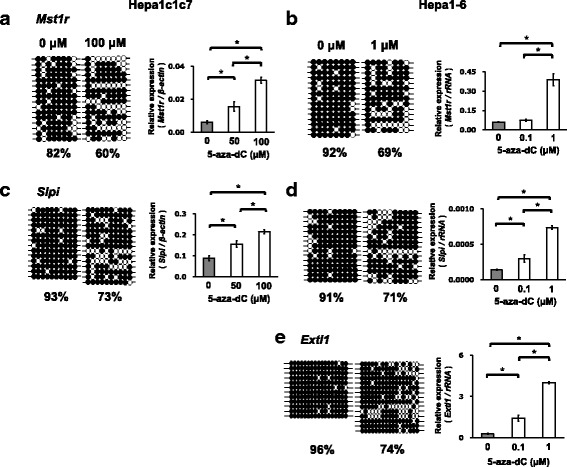
Reduced DNA methylation of DMRs of *Mst1r, Slpi,* and *Extl1* after 5-aza-dC treatment are associated with up-regulation of these genes in hepa1c1c7 cells and Hepa1-6 cells. Hepa1-6 cells (**b**, **d**, **e**) and Hepa1c1c7 cells (**a**, **c**) were cultured with 0, 0.1 or 1 μM of 5-aza-dC, and 0, 50 or 100 μM of 5-aza-dC for 72 h, respectively. Left figures: the results of bisulfite sequencing of CpGs detected in the DMRs of liver tumors in C3H mice. ●: methylated cytosine, ○: unmethylated cytosine. Right figures: the expressions of *Mst1r, Slpi,* and *Extl* were measured by real-time PCR and normalized to the expression of β-actin or rRNA (*n* = 3). Statistical significance was analyzed by one-way ANOVA followed by Turkey-Kramer test as a post hoc comparison. *^,^ **^,^ *** Significantly different at *p* < 0.05, 0.01, and 0.001, respectively

To determine whether DNA methylation alterations in the DMRs of *Mst1r, Slpi*, and *Extl1* are involved in the expression of these genes, we assessed the effects of the DNA methylation inhibitor 5-aza-dC on the DNA methylation statuses and gene expressions in mouse liver cancer cell lines (Fig. 5). *Mst1r* and *Slpi* were up-regulated and hypomethylated at DMRs by culturing Hepa1c1c7 cells with 50 μM and 100 μM of 5-aza-dC (Fig. 5a, c). Expression of Extl1 was not detected in Hepa1c1c7 cells. In Hepa1-6 cells, the three genes were up-regulated and their DMRs were hypomethylated by culturing with 0.1 and 1 μM of 5-aza-dC (Fig. 5b, d, e). These data support the notion that expressions of *Mst1r, Slpi* and *Extl1* are upregulated by hypomethylation of DMRs.

Interestingly, susceptibility against 5-aza-dC largely differed between the two cell lines. Measurement of gene expression levels of DNA methyltransferase *Dnmt1/3a/3b* and DNA demethylation enzymes T*et1/2/3,* the major enzymes determining DNA methylation level*,* clarified that Hepa1-6 cells have significantly lower expression levels of *Dnmts* in comparison with Hepa1c1c7 cells (Additional file 2: Figure S3). Expression levels of *Tet2* was also significantly lower in Hepa1-6 cells compared to Hepa1c1c7 cells (*p* < 0.01). *Tet1* expression was not detected in either cell line. These results suggest that the lowered expression of DNA methyltransferases is involved in the different sensitivity to 5-aza-dC between Hepa1-6 and Hepa1c1c7 cells.

### Transcription factor (TF) binding sites in the DMRs of *Mst1r* and *Slpi*

DNA methylation of TF binding sites or their proximity near the TSS can affect the binding of transcription factors and regulate transcription of genes [13, 30]. We searched for TF binding sites in the DMRs of *Mst1r, Slpi*, and *Extl1* using the JASPAR database [25] and literature. We found a CpG site overlapping NF-kB binding site in the DMR of *Mst1r* (Fig. 2a) and one overlapping AhR binding site in the DMR of *Slpi* (Fig. 3a). Methylation of CpG in the binding sequence for NF-kB [31] and AhR [32, 33] have been reported to inhibit their binding affinities. No CpG site overlapping the TF binding site was detected in the DMR of *Extl1* (Fig. 4a).

### Overexpression of *Mst1r* induces robust expression of *Il33*

We further assessed whether upregulation of *Mst1r* and *Slpi* play causal roles in tumorigenesis by overexpressing these genes and analyzing gene expression changes using microarrays in hepa1c1c7 cells.

We selected genes which are statistically significantly up or down regulated more than 10 fold by overexpression of *Mst1r* or *Slpi*, also show the same direction of significant expression changes in the liver tumors of C3H mice, and are found to be related with “cancer” by a PubMed search.

**Fig. 6:**
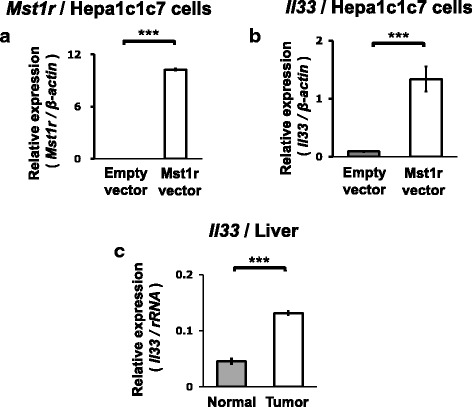
Overexpression of *Mst1r* induces IL33 in Hepa1c1c7 cells. Overexpression of *Mst1r* (**a**) and upregulation of *IL33* by *Mst1r* overexpression (**b**) in Hepa1c1c7 cells was confirmed by real-time PCR. *** Significantly different at *p* < 0.001 (*n* = 3). (**c**) Confirmation by real-time PCR of upregulation of *IL33* in the tumor tissues (*n* = 11) compared to the normal tissues (*n* = 6) of C3H mice. Statistical significance between the two groups was analyzed by the Student’s t-test. *** Significantly different at *p* < 0.001

Overexpression of *Mst1r* induced *IL33* which meets all the above criteria (Fig. 6a, b). Real-time PCR confirmed up-regulation of *IL33* in the liver tumors of C3H mice (Fig. 6c). No gene was identified under the criteria in the cells overexpressing *Slpi.*

### *Mst1r* and *Slpi* are upregulated in human HCC with altered DNA methylation status around the TSS

**Fig. 7:**
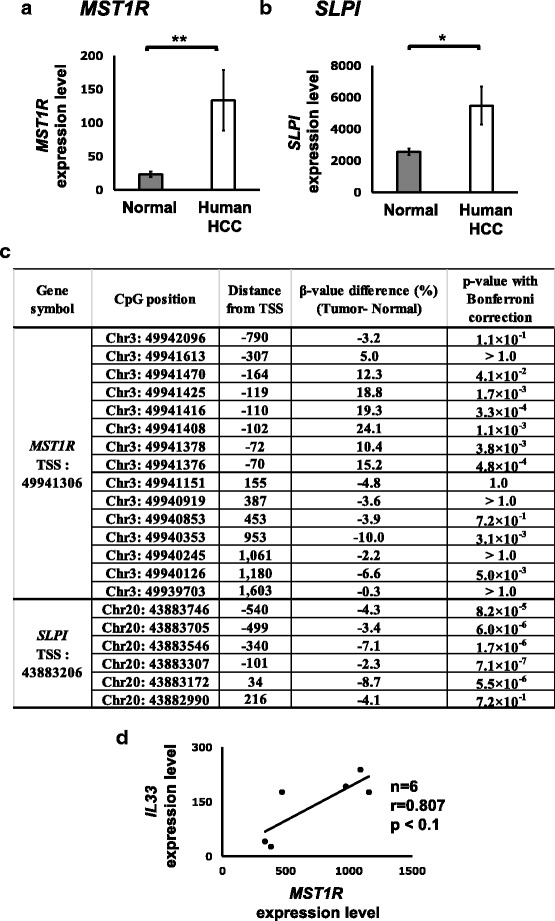
Human database searches showed hypomethylation of downstream regions of *MST1R* and *SLPI* with upregulation of their expressions. RNA-seq dataset and DNA methylation dataset of 41 paired normal and tumor tissues of human livers were downloaded from TCGA. **a**, **b** Average gene expression ratio (tumor tissues/normal tissues) was calculated using 41 paired data from TCGA. *^,^ ** Significantly different at *P* < 0.05, and 0.01, respectively. **c** The difference in DNA methylation β-value (tumor – normal) for each CpG around TSS was calculated using 41 paired data from TCGA. **d** The correlation between expressions of *MST1R* and *IL33* in the HCC tissues highly expressing *MST1R*

We analyzed the association between gene expression and DNA methylation status of human HCC by use of TCGA database. Data from 41 pairs of normal livers and HCC tissues were used for each analysis (Additional file 4: Table S3). Expression of *Mst1r* and *Slpi* are upregulated in the human HCC dataset we selected (Fig. 7a and b), as reported previously [27, 28]. Similar to the methylation status of *Mst1r* and *Slpi* in C3H mice, the downstream regions of TSS in both genes were weakly but significantly hypomethylated in human HCC compared to the normal liver tissues (Fig. 7c). These results showed the possibility that both the genes are regulated by DNA methylation, not only in the liver tumors of C3H mice, but also in human HCC. Furthermore, the expression of IL-33 strongly (*r* = 0.807), although not significantly (*p* < 0.1), correlated with *MST1R* expression in human HCC tissues highly expressing *MST1R* (Fig. 7d). Correlation was not observed between the expressions of the two genes when all the 41 pairs were analyzed (*r* = 0.095).

## Discussion

In the present study, we investigated the DNA methylation landscape of liver tumors of C3H mice by RRBS analysis. Hypo-DNA methylation is a well-known feature of tumors [34]. The present study clarified a large increase in the number of hypo-DMCs in the liver tumors of C3H mice (Fig. 1b), well illustrating the features of tumors. The present study further identified tumor-specific DMRs, including promoter DMRs for cancer-related genes.

Among the genes with promoter DMRs, we focused on three genes, *Mst1r*, *Slpi* and *Extl1* (Table 1). Mst1r activation is involved in tumor augmenting signaling pathways, such as the PI3K/AKT and RAS/ERK pathways [27]. *Slpi* is overexpressed in various types of cancers [28]. While there are studies reporting anti-metastatic activity of Slpi, a large body of studies reported overexpression of *Slpi* augments or associates with tumor proliferation and invasion in cancers of many origins [28, 35]. *Extl1* is reported as a putative tumor-suppressor gene [29]. The present study showed hypo-DMR around + 1000 bp from TSS and robust upregulation in *Mst1r* and *Slpi* in the tumors (Figs. 2 and 3) and hyper-DMR around + 1000 bp from TSS and downregulation of *Extl1* in the tumors (Fig. 4). These DMRs, which are not overlapped with CpG island, were not detected in our previous study on the spontaneous liver tumors of C3H mice using MeDIP-CpG microarrays [12]. Furthermore, this study firstly reported an association between expression changes of these genes and DMRs by an in vitro experiment using 5-aza-dC (Fig. 5).

We analyzed TCGA data for 41 pairs of normal and HCC tissues from patients and found overexpression of *MST1R* and *SLPI* and lowered DNA methylation of TSS down-stream region also in human HCC (Fig. 7). On the other hand, a recent study reported downregulation of *MST1R* and hypermethylation of the TSS upstream region in human hepatoblastomas [36]. The TSS upstream region of *MST1R* in the human HCC we analyzed was hypermethylated with transcription upregulation (Fig. 7c). Thus, *MST1R* expression and the association of DNA methylation status differ between HCC and hepatoblastomas. Aiming to target MST1R for cancer therapy, drugs such as tyrosine kinase inhibitors against the tyrosine kinase activity of MST1R and monoclonal antibodies against MST1R are being developed and some of these are under clinical trials or in research phases [27]. The results of the present study encourage further study on DNA methylation status around TSS of *Mst1r* to develop epigenetic therapies.

In the present study, we further identified *IL33* as the putative down-stream targets of Mst1r by overexpression of *Mst1r* into hepa1c1c7 cells (Fig. 6c). *IL33* was also upregulated in the tumor tissues of C3H mice (Fig. 6e) and its expression is associated with *MST1R* expression in higher *MST1R* expressing group (Fig. 7d). *IL33* activates NF-kB and ERK signaling pathways and is implicated in HCC [37]. The present study newly demonstrates the possibility that *IL33* is a down-stream target of Mst1r and is involved in the liver tumors of C3H mice and in human HCC.

DNA methylation exerts a variety of modes of action. DNA methylation of TF binding sequences affects the affinity of TFs to DNA [13, 30]. Hypermethylation of the *Fas* promoter, which contains 3 putative NF-kB binding sites inhibits NF-kB binding [31]. NF-kB pathway is deeply involved in HCC in human [38] as well as C3H mice [39, 40]. The DMR of *Mst1r* in the C3H mice tumors contained the putative NF-kB consensus sequence (5′- GTGGAGCCCC(G)-3′) and the CpG in the sequence is hypomethylated (Fig. 2b). Thus, hypomethylation of CpGs in the putative NF-kB site in the DMR of *Mst1r* may take a part in augmenting NF-kB binding and activating *Mst1r*. The DMR of *Slpi* includes the core sequence (5’-CGTG-3′) of xenobiotic-responsible element (XRE) and its hypomethylation inhibits the binding of AhR/Arnt complex [32, 33]. Thus, hypermethylation of CpG in the XRE core sequence in *Slpi* (Fig. 3b) may be involved in *Slpi* activation through AhR/Arnt binding, while the involvement of AhR/Arnt in spontaneous hepatic tumors is unknown. Contribution of the methylation status in TF binding sites require further studies.

During this study, we found that the sensitivity of Hepa1c1c7 cells and Hepa1-6 cells to 5-aza-dC is largely different (Fig. 5). 5-aza-dC is now utilized as epigenetic medicine to recover the expression of tumor suppressor genes by reducing DNA methylation [41]. 5-Aza-dC is transported into cells by nucleoside transporters, incorporated into DNA after being triphosphorylated by deoxy-cytidine kinase, and inhibits DNMT1 activity by forming a covalent bond with the enzyme or augmenting DNMT1 degradation. We measured the expression of DNA methylation and demethylation enzymes and found significantly lower expression of *Dnmt1*, *Dnmt3a* and *Dnmt3b* in Hepa1-6 cells compared to Hepa1c1c7 cells. Thus the suppression of *Dnmts* may contribute to the difference in the sensitivity of the cells to 5-aza-dC.

## Conclusions

The present study clarified the DNA methylation landscape in association with gene expression in the liver tumors of C3H mice. The data provide useful information for further genome-wide studies of mice models of HCC. The present study particularly proposed novel pathways regulated by DNA methylation for *Mst1r* and *Slpi*, which may be applied not only to mouse liver tumors but also to human HCC.

## Additional files

Additional file 1: **Table S1.** Primers for bisulfite sequencing and qRT-PCR. (XLSX 10 kb)

Additional file 2: **Figure S1.** Scatter plots for sample pairs. Scatter plots of % methylation values for each pair in normal tissues (*n* = 3) and tumor tissues (*n* = 3) were obtained using methylKit. Numbers on upper right corner denote pair-wise Pearson’s correlation scores. The histograms on the diagonal are methylation distribution of CpG sites for each sample. **Figure S2/.** Expression levels of *Mst1r, Slpi and Extl1* in tumor tissues with (*n* = 6) and without (*n* = 5) Ha-ras mutation. **Figure S3.** Hepa1-6 cells show lower expression of Dnmts compared to Hepa1c1c7. cDNA was prepared from Hepa1c1c7 cells and Hepa1-6 cells and the expression was measured by real-time PCR (*n* = 3). All genes were normalized to the expression of *rRNA*.. *** *p* < 0.001. (PPTX 296 kb)

Additional file 3: **Table S2.** List of DMRs position and methylation difference between normal and tumor liver tissues. (XLSX 317 kb)

Additional file 4: **Table S3.** Datasets of DNA methylation and gene expression downloaded from TCGA database. (XLSX 13 kb)

## Abbreviations

5-aza-dC: 5-aza-2′-deoxycytidine
CCl4: Carbon tetrachloride
DEN: N-nitrosodiethylamine
DMC: Differentially methylated cytosine
DMEM: Dulbecco’s modified Eagle’s medium
DMR: Differentially methylated region
FBS: Fetal bovine serum
FLC: Fibrolamellar hepatocellular carcinoma
HCC: Hepatocellular carcinoma
MeDIP: Methylated DNA immunoprecipitation
ORF: Open reading frame
RBSS: Reduced representation bisulfite sequencing
TCGA: The Cancer Genome Atlas
TF: Transcription factor
TSS: Transcription start site
