# Supplementary material for: The DNA methylation profile of liver tumors in C3H mice and identification of differentially methylated regions involved in the regulation of tumorigenic genes
Source: BMC Cancer. 2018 Mar 22;18:317. doi: 10.1186/s12885-018-4221-0 (PMC5865360; doi:10.1186/s12885-018-4221-0)
Supplement: Supplementary file 2 — Figure S1. Scatter plots for sample pairs. Scatter plots of % methylation values for each pair in normal tissues (n = 3) and tumor tissues (n = 3) were obtained using methylKit. Numbers on upper right corner denote pair-wise Pearson’s correlation scores. The histograms on the diagonal are methylation distribution of CpG sites for each sample. Figure S2/. Expression levels of Mst1r, Slpi and Extl1 in tumor tissues with (n = 6) and without (n = 5) Ha-ras mutation. Figure S3. Hepa1-6 cells show lower expression of Dnmts compared to Hepa1c1c7. cDNA was prepared from Hepa1c1c7 cells and Hepa1-6 cells and the expression was measured by real-time PCR (n = 3). All genes were normalized to the expression of rRNA.. *** p < 0.001. (PPTX 296 kb) [file 12885_2018_4221_MOESM2_ESM.pptx]

## Slide 1
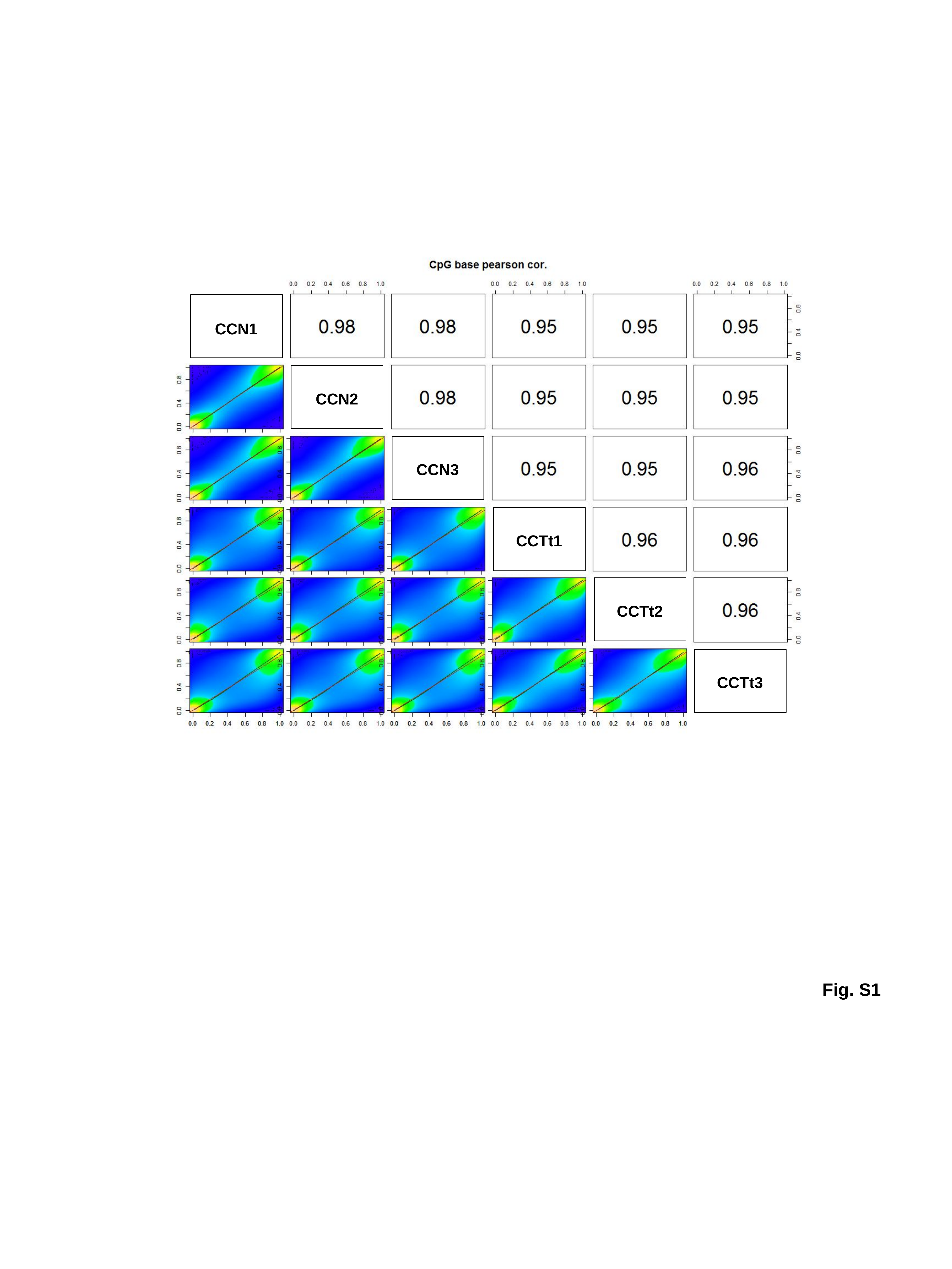

CCN1
CCN2
CCN3
CCTt1
CCTt2
CCTt3
Fig. S1

## Slide 2
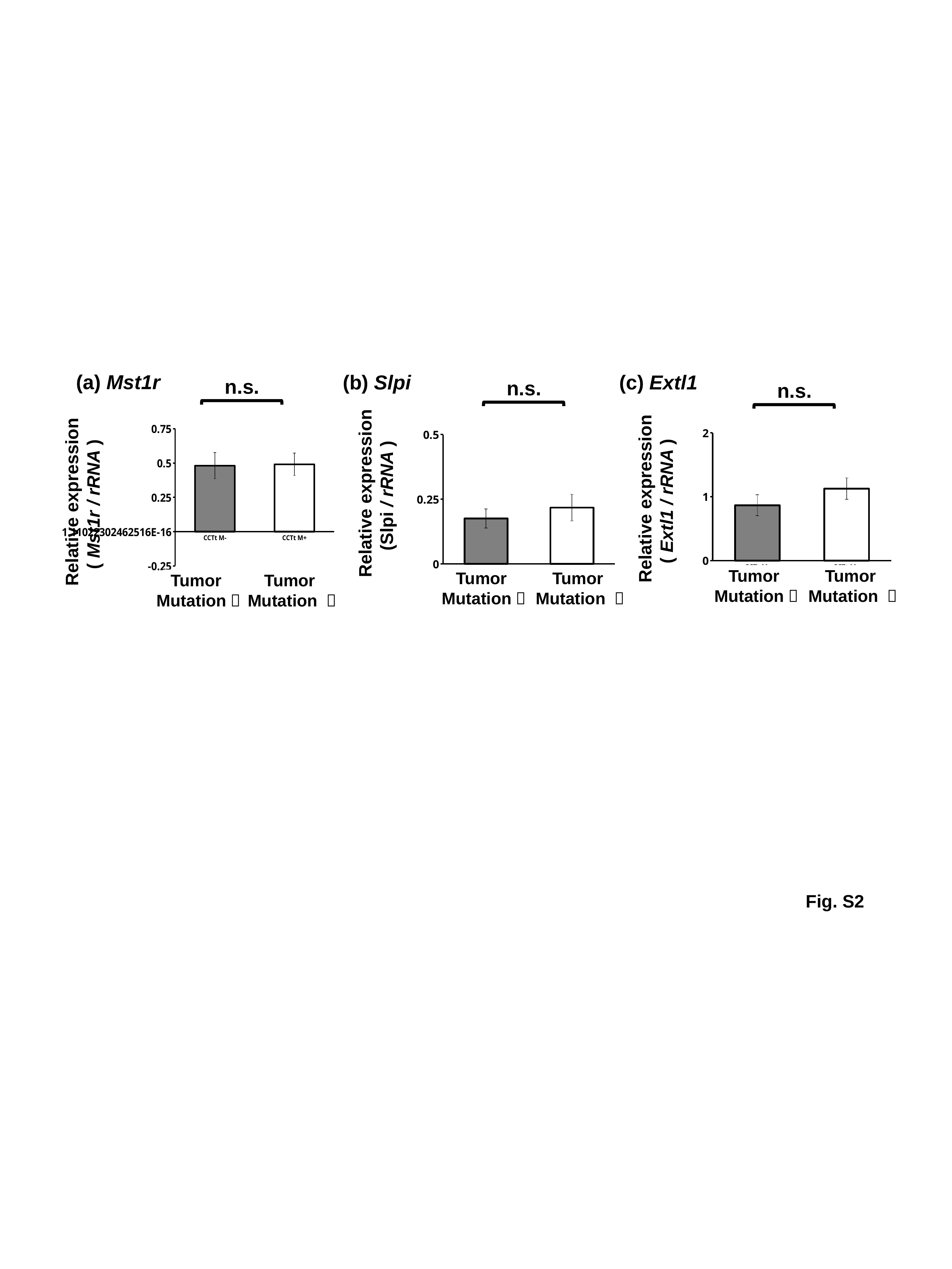

(a) Mst1r
(b) Slpi
(c) Extl1
n.s.
n.s.
n.s.
Relative expression
(Slpi / rRNA )
Relative expression
( Extl1 / rRNA )
Relative expression
( Mst1r / rRNA )
### Chart
| Category | |
|---|---|
| CCTt M- | 0.4811967183739605 |
| CCTt M+ | 0.491042539031897 |
### Chart
| Category | |
|---|---|
| CCTt M- | 0.17543743075641838 |
| CCTt M+ | 0.21760873864600852 |
### Chart
| Category | |
|---|---|
| CCTt M- | 0.8667372395137258 |
| CCTt M+ | 1.1267695881517894 |Tumor
Mutation－
Tumor
Mutation ＋
Tumor
Mutation－
Tumor
Mutation ＋
Tumor
Mutation－
Tumor
Mutation ＋
Fig. S2

## Slide 3
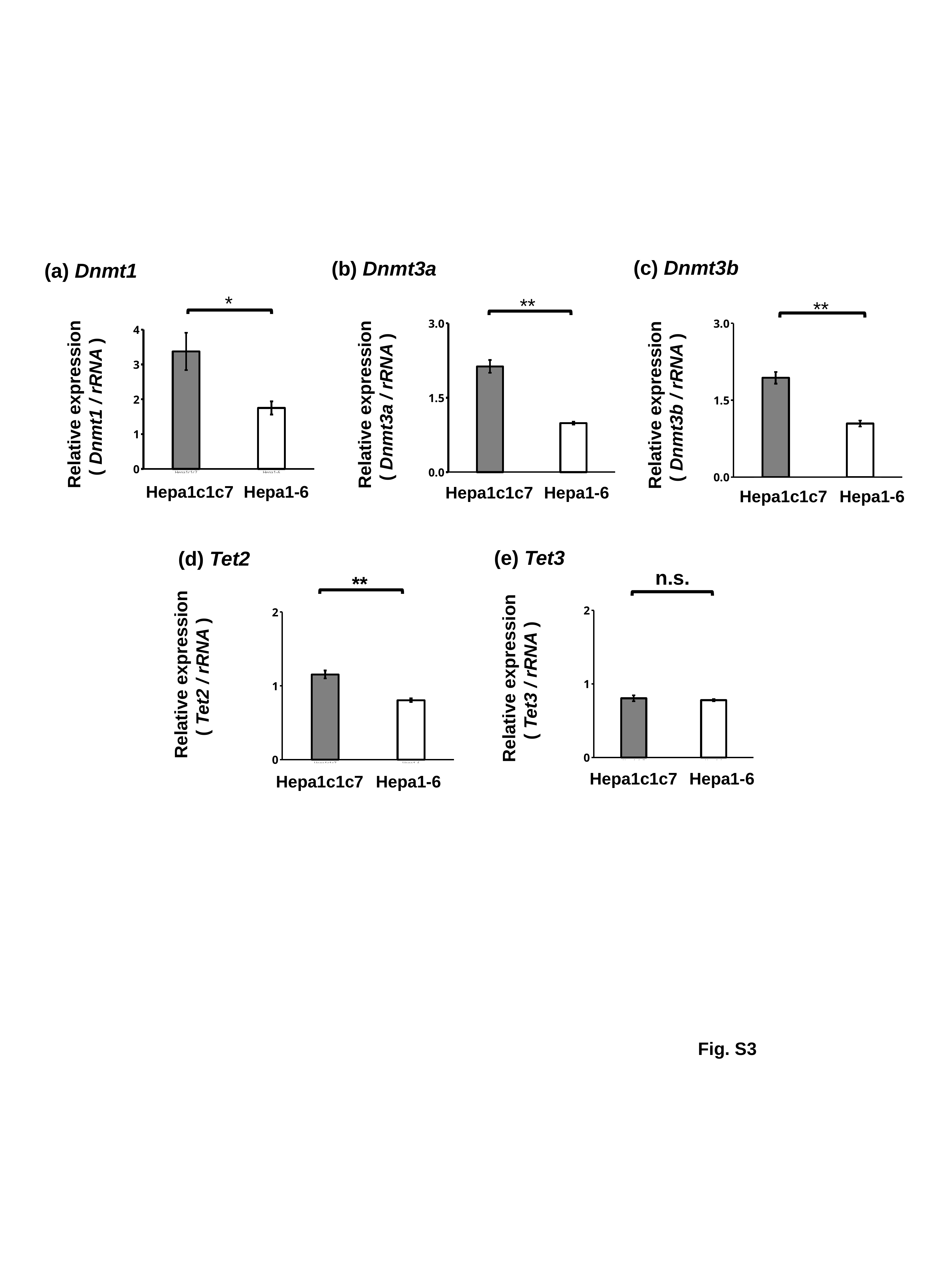

(c) Dnmt3b
(b) Dnmt3a
(a) Dnmt1
*
**
**
### Chart
| Category | |
|---|---|
| Hepa1c1c7 | 2.1304249511054265 |
| Hepa1-6 | 0.9875808678340668 |
### Chart
| Category | |
|---|---|
| Hepa1c1c7 | 3.3750160638838795 |
| Hepa1-6 | 1.7506183163641476 |
### Chart
| Category | |
|---|---|
| Hepa1c1c7 | 1.936705992927905 |
| Hepa1-6 | 1.0440797198000586 |Relative expression
( Dnmt1 / rRNA )
Relative expression
( Dnmt3a / rRNA )
Relative expression
( Dnmt3b / rRNA )
Hepa1-6
Hepa1c1c7
Hepa1-6
Hepa1c1c7
Hepa1-6
Hepa1c1c7
(e) Tet3
(d) Tet2
n.s.
**
Relative expression
( Tet2 / rRNA )
Relative expression
( Tet3 / rRNA )
### Chart
| Category | |
|---|---|
| Hepa1c1c7 | 1.1537096849803499 |
| Hepa1-6 | 0.8047370314617363 |
### Chart
| Category | |
|---|---|
| Hepa1c1c7 | 0.8042244564108388 |
| Hepa1-6 | 0.7791561657792748 |
Hepa1-6
Hepa1c1c7
Hepa1-6
Hepa1c1c7
Fig. S3
